# Quercetin‑conjugated superparamagnetic iron oxide nanoparticles modulate glucose metabolism-related genes and miR-29 family in the hippocampus of diabetic rats

**DOI:** 10.1038/s41598-021-87687-w

**Published:** 2021-04-21

**Authors:** Solmaz Dini, Mansoureh Zakeri, Shiva Ebrahimpour, Fariba Dehghanian, Abolghasem Esmaeili

**Affiliations:** Department of Cell and Molecular Biology & Microbiology, Faculty of Biological Science and Technology, University of Isfahan, Isfahan, Iran

**Keywords:** Drug discovery, Molecular biology, Neuroscience

## Abstract

Quercetin (QC) is a dietary bioflavonoid that can be conjugated with nanoparticles to facilitate its brain bioavailability. We previously showed that quercetin-conjugated superparamagnetic iron oxide nanoparticles (QCSPIONs) reduced the level of blood glucose in diabetic rats. Glucose transporters (GLUTs), insulin-like growth factor-1 (IGF-1), and microRNA-29 (miR-29) play a critical role in brain glucose homeostasis. In the current study, we examined the effects of QCSPION on the expression of glucose metabolism-related genes, and the miR-29 family as a candidate regulator of glucose handling in the hippocampus of diabetic rats. Our in silico analyses introduce the miR-29 family as potential regulators of glucose transporters and IGF-1 genes. The expression level of the miR-29 family, IGF-1, GLUT1, GLUT2, GLUT3, and GLUT4 were measured by qPCR. Our results indicate that diabetes significantly results in upregulation of the miR-29 family and downregulation of the GLUT1, 2, 3, 4, and IGF-1 genes. Interestingly, QCSPIONs reduced miR-29 family expression and subsequently enhanced GLUT1, 2, 3, 4, and IGF-1expression. In conclusion, our findings suggest that QCSPION could regulate the expression of the miR-29 family, which in turn increases the expression of glucose transporters and IGF-1, thereby reducing diabetic complications.

## Introduction

Diabetes mellitus (DM) is the most prevalent disorder of the endocrine system which is recognized by hyperglycemia, resulting from defects in the secretion or/and function of insulin^1^. It has been shown that hyperglycemic conditions can strongly affect brain function. On the other hand, hypoglycemic shock induced by diabetes can lead to the central nervous system (CNS) dysfunction and brain cell death^2^. Glucose is the primary source of metabolic energy for the CNS and it goes across the plasma membrane of neurons with the help of glucose transporters (GLUTs)^3,4^. GLUT1, 2, 3, and 4 are widely distributed in the CNS, especially in the hippocampus, and play a vital role in brain glucose homeostasis^5,6^. GLUT1 is the main responsible for glucose transport from the blood–brain barrier (BBB) which is an insulin-independent and glucose insensitive GLUT. GLUT3 controls glucose absorption into the neuron^7^. Besides, the wide distribution of GLUT4, insulin and insulin-like growth factor-1 (IGF-1) receptors on the surface of neurons and glial cells showed that the brain is an insulin-sensitive organ^8^. A wide range of studies have shown that the expression, regulation, and activity of GLUTs can be disrupted during hyperglycemia^4,9,10^. These changes negatively affect glucose metabolism in the brain which results in impairing of synaptic plasticity, neurogenesis, and cognitive function^11,12^. Moreover, downregulation of neurotrophic factors such as IGF-1 was observed in the diabetic brain, which contributes to diabetic neurological disorders^13,14^. IGF-1 is a polypeptide hormone that is structurally and functionally homologous to insulin^15,16^. The biological activity of IGF-1 in the brain is done through its specific receptor (IGF-1R), which is expressed in different parts of the brain, including the hippocampus^17^. Like insulin, IGF-1 stimulates glucose absorption and metabolism in the brain. Interaction of IGF-1 with its receptor in different parts of the brain triggers the PI3K/Akt signaling pathway. PI3K/Akt signaling leads to translocation of GLUT4 to neuronal cell membranes, promoting glucose uptake into neurons^18,19^.

The role of microRNAs has been studied in diabetes and its complications^20^. Studies have highlighted the role of the microRNA-29 (miR-29) family in glucose homeostasis^21^. miR-29 family with three mature members of miR-29a, miR-29b, and miR-29c is a sensitive microRNA family to insulin deficiency and hyperglycemia^22,23^. The miR-29 family members with identical seed sequences target largely overlapping sets of genes^24^. Bioinformatics studies on the expression profile of miRNAs in diabetes show that GLUT1-4 and IGF-1 mRNAs are potential targets of the miR-29 family. Several studies have shown that GLUT1-4 and IGF-I genes are direct targets of the miR-29 family using different methods including in silico analyses, luciferase assay and qRT-PCR^23,25–29^. Studies in diabetic patients and animal models revealed that the expression of miR-29 family is increased in different tissues, including liver, pancreas, kidney, skeletal muscle, adipose tissue, and brain^30–37^. It is well known that miR-29 family negatively regulates the expression of GLUTs and IGF-1 in diabetic conditions^23,29,38–40^. Collectively, miR-29 family members can be considered as early markers of DM^41^.

Since the treatment of diabetes with chemical drugs have serious side effects, it is necessary to introduce exogenous antioxidants supplements, especially in the early stage of diabetes^14^. In this context, quercetin (QC) with the chemical formula C15H10O7 is identified as one of the most abundant flavonoid polyphenolic molecules in many fruits, vegetables, and seeds^42^. QC has beneficial effects on various diseases, such as cardiovascular disease, cancer, infection, diabetes, obesity, and neurological disorders^43^. There is strong evidence that QC improves hyperglycemia through a direct effect on gene expression at the transcriptional level and also by modulation of miRNAs as a part of the post-transcriptional gene regulation^44^. Although QC has multiple medicinal benefits, its low aqueous solubility, instability in the physiological environment, and poor bioavailability have restricted the use in clinical application^45^. Therefore, to overcome these limitations, it is necessary to develop a system that could protect QC against enzymatic degradation and provide an elevated pool of it in the body tissues, especially in the CNS^42^. Superparamagnetic iron oxide nanoparticles (SPIONs) are well accepted as an effective drug delivery system because of their unique size, colloidal stability, crossing the BBB, bio-distribution, and good safety profile^46^. Besides, SPIONs are magnetic targeted carriers (MTC) that can be localized in certain tissue under an external magnetic field. Currently, there are multiple FDA-approved magnetite-based nanoparticles, as well as numbers of applicable SPIONs are ongoing^47^. Application of the quercetin-conjugated superparamagnetic iron oxide nanoparticles (QCSPIONs) in animal models and cell culture has led to considerable results in our previous studies. In this regard, we reported that exposing PC12 cells to QCSPIONs (10 − 100 μg mL^−1^) caused a remarkable outgrowth of neurite and enhanced the neuronal branching complexity better than pure QC^48^. We reported that the antitoxic activity of SPIONs such as the catalase-like activity remained unchanged after conjugation in H2O2-induced toxicity condition of PC12 cells^49^. We also suggested that the concentration of QC in the brains of QCSPION-treated healthy rats was about 4.8 times for 50 mg kg^−1^ of QC and 8.6 times for 100 mg kg^−1^ of QC higher than rats treated with pure QC. Therefore, it can be concluded that SPIONs improve the bioavailability of QC and its passage through the BBB^50^. Besides, we showed that treatment with both QC and QCSPIONs (50 mg kg^−1^ and 100 mg kg^−1^) had no effect on hepatic GSH, TAC, MDA levels, and CAT activity of healthy rats^51^. In another study, we showed that treatment with QCSPIONs (50 and 100 mg kg^−1^) during 1 week improved memory performance in healthy rats better than pure QC via their interaction with proteins involved in Long-Term Potentiation (LTP)^52^. Because of the causal role of diabetes in CNS disorders especially cognitive dysfunction and dementia, we used QCSPIONs to reduce the effects of diabetes on learning and memory. We showed that oral delivery of QCSPIONs (25 mg kg-1) reduced blood glucose level and ameliorate learning and memory impairment of diabetic rats^53^. We focused on inflammation and oxidative stress as the underlying molecular mechanisms of these effects and reported that QCSPIONs could improve learning and memory impairment through targeting NF-κB/miR-146a and Nrf2/miR-27a pathways^54,55^. In the current study, we targeted GLUTs (GLUT1, GLUT2, GLUT3, and GLUT4), IGF-1, and miR-29 family as other classical targets of QCSPIONs in diabetic condition. Although several experimental studies have shown the role of GLUTs, IGF-1, and miR-29 family in the pathogenesis of diabetic complications, there is no enough knowledge about the effect of QC and QCSPIONs on the modulation of these genes. Thus, the purpose of the current study was to compare the effect of QC and QCSPIONs on the expression of GLUT1, GLUT2, GLUT3, GLUT4, and IGF-1 genes, as well as miR-29 family in the hippocampus of diabetic rats.

## Results

### In silico analyses introduce the miR-29 family as a potential regulator of GLUTs and IGF-1

**Figure 1:**
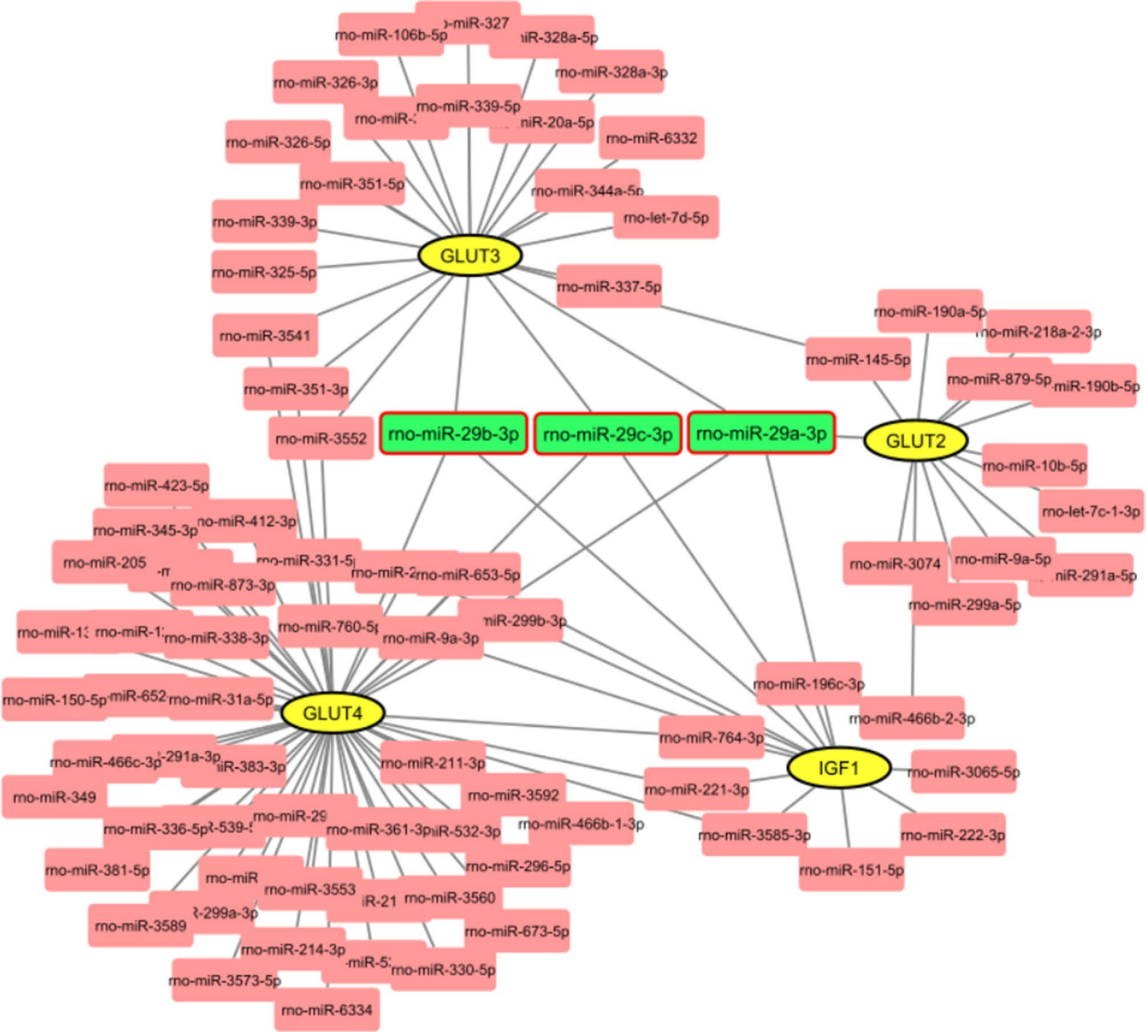
Potential regulation of glucose transporters and IGF-1 by miR-29 family. Among all miRNAs which target the GLUT1, 2, 3, 4, and IGF-1 genes, three members of the miR-29 family can target commonly all GLUT3, 4, and IGF-1 genes. GLUT2 is also targeted by miRNA-29a. Cityscape 3.8.0 (https://cytoscape.org/release_notes_3_8_0.html) was used for network representation.

At the first step, we prepare a list of miRNAs that are involved in DM through a comprehensive literature review (Table 1)^56–59^. The miRNAs which were reported in different tissues relating to type 1 diabetes (T1D) at least in two studies were selected. Then, miRWalk 2.0 (http://zmf.umm.uni-heidelberg.de/apps/zmf/mirwalk2/custom.html), a target prediction resource that uses several miRNA target prediction tools and TargetScan 7.2 (http://www.targetscan.org/vert_72/) were used for prediction of miRNAs which target the 3′UTR region of GLUT1, 2, 3, 4 and IGF-1 genes. The analysis was limited to the target 3′UTR region of GLUT2, 3, 4, and IGF-1 genes of the rat because despite human GLUT1 data, no data is referring to rat GLUT1 gene in two databases. Our results indicate that GLUT 3, 4, and IGF-1 genes are targeted by miRNA-29a, miRNA-29b, and miRNA-29c. Furthermore, miRNA-29a can target the GLUT2 gene (Fig. 1). Finally, three miRNAs of the miR-29 family including, miRNA-29a, miRNA-29b, and miRNA-29c were filtered for further experimental investigations concerning their roles in diabetes and targeting four target genes.

**Table 1:** List of important miRNAs involved in DM.

miRNA	Target of gene	Change	Organ/cell type	Reference
miR-34a	Foxp1	Upregulation	NOD.B10 Idd9.3 mice	^85^
miR-30b	Neurod1	Upregulation	Inflammatory cytokine-mediated_ cells	^86^
miR-21	Bcl-2	Upregulation	NOD mice and mice treated with streptozotocin, INS-1 832/13_ cells, and human islets	^87^
miR-326	Vitamin D receptor and erythroblastosis virus E26	Upregulation	Peripheral blood lymphocytes ofT1D patients	^88^
miR-141	Slc25a3	Upregulation	HL-1 cells and heart of T1D mice	^89^
miR-29b/c	GLUT4	Upregulation	Muscle of rats	^23^
miR-29 a/c	GLUT1	Upregulation	Primary human skeletal muscle cells	^38^
miR-29a/b	GLUT2	Upregulation	The pancreas of NONcNZO10/LtJ mice	^29^
miR-29a	IGF-1	Upregulation	Myocardial cells	^39^
miR-29	IGF-1	Upregulation	Myocardial microvascular endothelial cells	^27^
miR-145	ABCA1 and IGFR1	Downregulation	Rat with T1DM	^90^
miR-144	Nrf2	Downregulation	Streptozotocin-induced diabetic mice and cultured cardiomyocytes	^91^
miR-146a	TRAF6, STX3, BCL11A, and NUMB	Downregulation	PBMC	^92^
miR-23a-3p, miR-23b-3p	Proapoptotic BH3-only proteins DP5 and PUMA	Downregulation	Human pancreatic β cells	^93^

### QC and QCSPIONs significantly reduce the expression of the miR-29 family in the hippocampus

**Figure 2:**
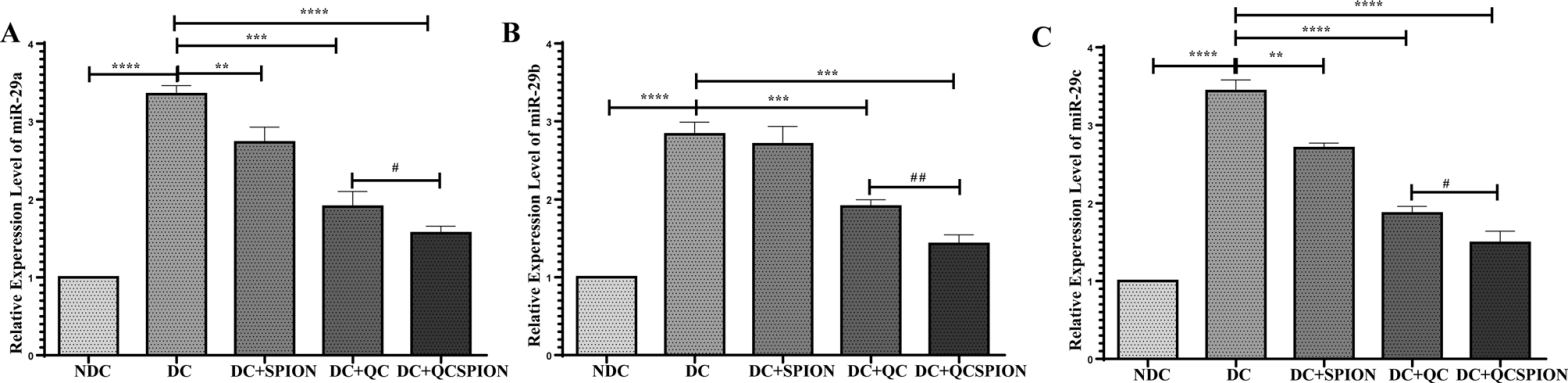
Effect of QC and QCSPIONs treatment on miR-29 family. (**A**) miR-29a, (**B**) miR-29b, (**C**) miR-29c in the hippocampus of diabetic rats. Data are mean with SEM (n = 5 per group). **P < 0.01, ***P < 0.001 and P < 0.0001 vs DC group (One-way ANOVA, Tukey’s multiple comparison tests). ^#^P < 0.05, ^##^P < 0.01 vs QCSPION group. *NDC* non-diabetic control, *DC* diabetic control, *DC + SPION* diabetic treated with superparamagnetic iron oxide nanoparticle, *DC + QC* diabetic treated with quercetin, *DC + QCSPION* diabetic treated with quercetin-conjugated superparamagnetic iron oxide nanoparticle.

The expression levels of the miR-29 family (miR-29a, miR-29b, miR-29c) in the hippocampus of the studied groups are shown in Fig. 2. One-way ANOVA analysis indicated that expression levels of miR-29a and miR-29c were increased more than threefold and miR-29b more than twofold in the diabetic group (p < 0.0001 for miR-29a, b, c). Treatment with pure and conjugated forms of QC significantly reduced the expression level of miR-29a (p < 0.001 for Q and p < 0.0001 for QCSPIONs), miR-29b (p < 0.001 for Q and QCSPIONs), and miR-29c (p < 0.0001 for Q and QCSPIONs) in the hippocampus of diabetic rats. What is interesting in this data is that the most significant effect was observed in the QCSPIONs treated group so that the expression levels of miR-29 family in QCSPIONs rats were significantly lower than those that received pure QC.

### QC and QCSPIONs significantly increase the expression of GLUTs in the hippocampus

**Figure 3:**
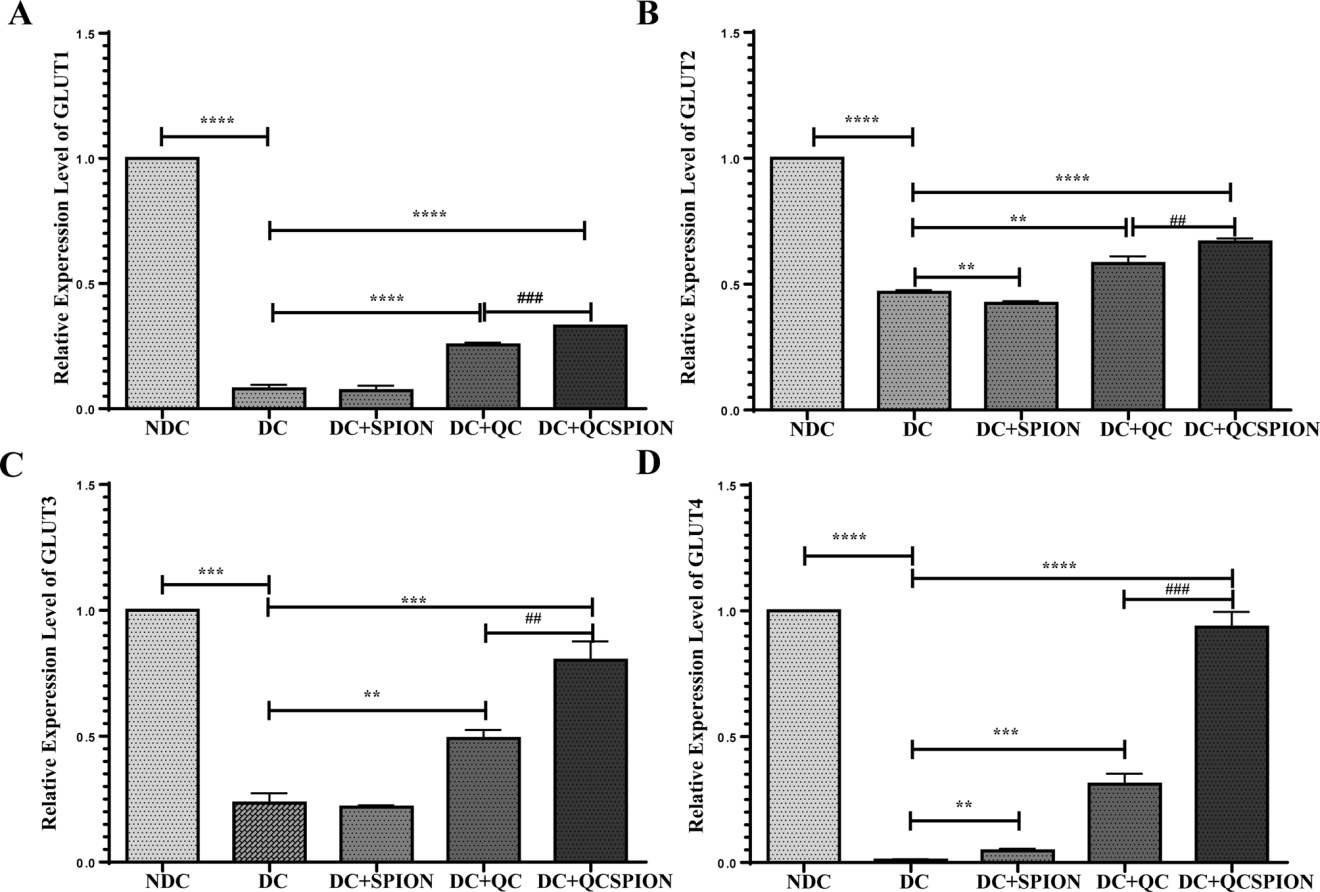
Effect of QC and QCSPIONs treatment on mRNA levels of glucose transporters. (**A**) GLUT1, (**B**) GLUT2, (**C**) GLUT 3, (**D**) GLUT4 in the hippocampus of diabetic rats. Data are mean with SEM (n = 5 per group). **P < 0.01, ***P < 0.001 and P < 0.0001 vs DC group (One-way ANOVA, Tukey’s multiple comparison tests). ^##^P < 0.01, ^###^P < 0.001 vs QCSPION group. *NDC* non-diabetic control, *DC* diabetic control, *DC + SPION* diabetic treated with superparamagnetic iron oxide nanoparticle, *DC + QC* diabetic treated with quercetin, *DC + QCSPION* diabetic treated with quercetin-conjugated superparamagnetic iron oxide nanoparticle, *GLUT* glucose transporter.

Real-time PCR results derived from mRNA expression levels of GLUT1, GLUT2, GLUT3, and GLUT4 in the hippocampus of diabetic rats are represented in Fig. 3. The expression levels of GLUT1, GLUT2, GLUT3, and GLUT4 in the hippocampus of diabetic rats were decreased significantly compared to the control group (P < 0.0001 for GLUT1-4). As shown in Fig. 3, after treatment with QC, expression levels of all studied GLUTs were increased significantly (P < 0.0001 for GLUT1, P < 0.01 for GLUT2, P < 0.01 for GLUT3, and P < 0.0001 for GLUT4). Moreover, we found that QCSPIONs were able to increase the expression of GLUT2, GLUT3, and GLUT4 up to a normal level more effectively than pure QC (P < 0.0001 for GLUT2, P < 0.001 for GLUT3, and P < 0.0001 for GLUT4). What is interesting in these results is that expression levels of all studied GLUTs in QCSPIONs rats were significantly higher than those that received pure QC.

### QC and QCSPIONs significantly increase the expression of IGF-1 in the hippocampus

**Figure 4:**
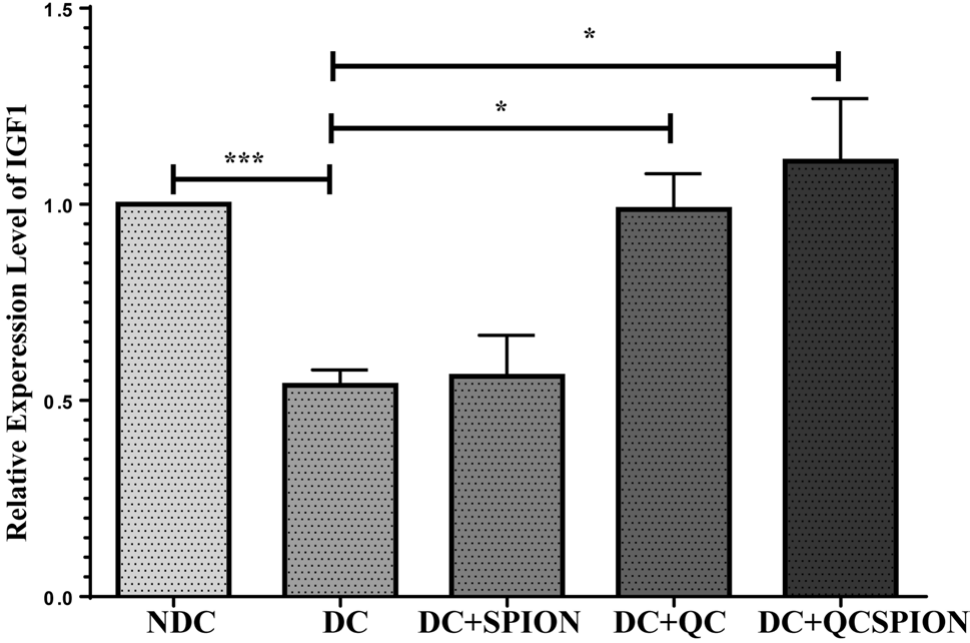
Effect of QC and QCSPIONs treatment on mRNA level of IGF-1 in the hippocampus of diabetic rats. Data are mean with SEM (n = 5 per group). *P < 0.05, ***P < 0.001 vs DC group (One-way ANOVA, Tukey’s multiple comparison tests). *NDC* non-diabetic control, *DC* diabetic control, *DC + SPION* diabetic treated with superparamagnetic iron oxide nanoparticle, *DC + QC* diabetic treated with quercetin, *DC + QCSPION* diabetic treated with quercetin-conjugated superparamagnetic iron oxide nanoparticle.

As shown in Fig. 4, the expression level of IGF-1 was decreased significantly in the hippocampus of diabetic rats compared to the control group (P < 0.001). The comparison between groups showed that treatment with QC and QCSPIONs led to a significant increase in IGF-1 mRNA expression level compared to the diabetic group (P < 0.05 for Q and QCSPIONs).

## Discussion

**Figure 5:**
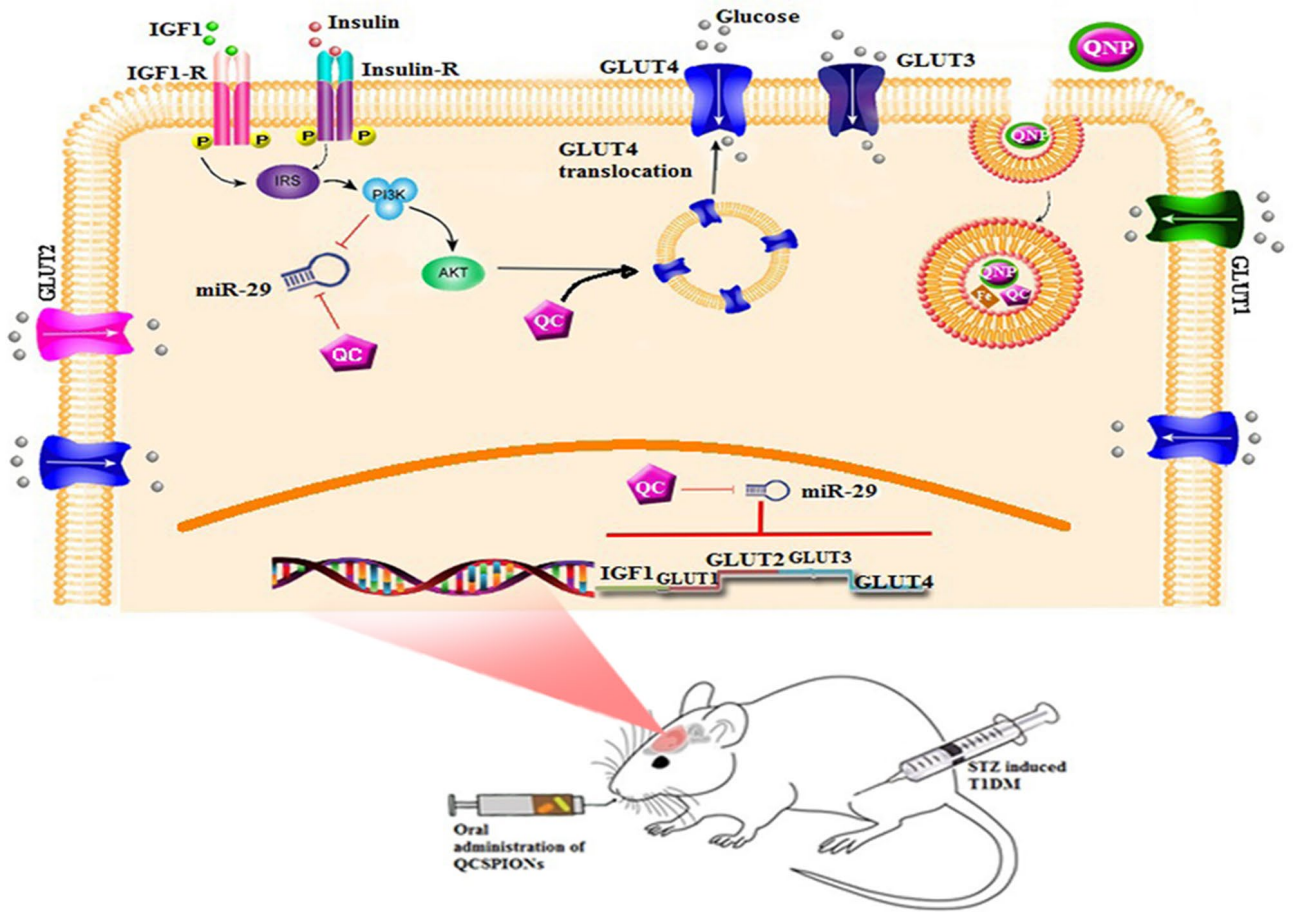
Schematic image of the protective effect of QC after released from superparamagnetic iron oxide nanoparticles on metabolism-related genes and miR-29 family in the hippocampus of streptozotocin-induced diabetic rats. This Figure was drew by Mansoureh Zakeri using powerPoint and Paint.

Diabetes causes secondary pathophysiological changes in the brain and diabetic patients are significantly more likely to develop cognitive impairment. QC has the potential to treat glucose metabolism disorders in DM and has been considered as a promising drug combination to reduce diabetes complications^43^. In a previous report, we showed that dextran-coated superparamagnetic iron oxide nanoparticles with minimal toxicity improve learning and memory impairment in diabetic rats better pure QC even at concentrations below pure QC^53^. The goal of the current study is assessment of glucose metabolism-related genes and the miR-29 family as one of the classical targets of QCSPIONs in diabetic conditions (Fig. 5).

First, miRNA-mRNA interaction of glucose transporters genes,IGF1 gene and miR-29 family was predicted. Then, the anti-diabetic and neuroprotection effects of QC and QCSPION on the expression of miR-29 family in the hippocampus of diabetic rats were detected. Real-time PCR results show that the expression levels of miR-29a and miR-29c in the hippocampus of diabetic rats were increased more than threefold and miR-29b more than twofold in comparison to the control group. Several studies have shown that the expression of miR-29 family is increased by hyperglycemia and proinflammatory cytokines, which are two main hallmarks of diabetes^41^. Upregulation of miR-29 is a key factor in the loss of pancreatic beta cells and development of the first stage of T1D^41^. As we reported in our previous study, induction of diabetes for fifty days destroyed pancreatic beta cells and significantly increased blood glucose level in rats. Besides, we showed that levels of inflammatory cytokines such as TNF-α increased in the hippocampus of diabetic rats^54^. Therefore, it can be concluded that increased expression of miR-29 can be due to hyperglycemia and the presence of inflammatory markers. These findings are in agreement with studies conducted on cell cultures and diabetic models that indicated upregulation of miRNA-29a-c in different tissues. In this regard, a study by Li et al. in 2017 showed that inhibition of IGF-1 expression by the miR-29 family led to myocardial apoptosis and cardiovascular complications in diabetic rats^27^. Massart et al. in 2017 reported that overexpression of miR-29a and miR-29c in cultured human skeletal muscle primary cells impaired glucose metabolism by decreasing GLUT1 expression^38^. Hirata et al. in 2017 showed that increased expression of miR-29a and miR-29b led to downregulation of GLUT2 expression in the pancreas of NONcNZO10/LtJ mice^29^. The result of a study conducted by Esteves et al. in 2018 showed that upregulation of miR-29b-3p and miR-29c-3p led to reduction of GLUT4 expression by 50 to 77% in the skeletal muscle of diabetic rat^23^.

Our results indicate that treatment with QC in both pure and conjugated forms normalized the upregulation of miR-29 family. Despite several studies regarding the modulation effect of QC on miRNA expression in different pathological conditions^60^, no study has been investigated the effect of this flavonoid on miR-29 family expression. However, it can be proposed that the reduced expression of miR-29 family is the result of modulation effect of QC on miRNAs expression as a part of the post-transcriptional gene regulation and direct effect of QC on the reduction of hyperglycemia and proinflammatory cytokines, and regeneration of islets of Langerhans^61,62^. Anti-diabetic effects of QC in pure and it's compound with other agents have been reported through the regeneration of the pancreatic islets, increasing insulin secretion, inhibition of Glucose-6-phosphatase, augmenting liver glycogen content, and stimulation of GLUT expression and translocation^43,63^.

In the current study, GLUTs mRNA levels were decreased in the hippocampus of diabetic rats. It is well studied that majority of glucose absorption in the hippocampus is mediated through GLUT1 which is expressed in microvessels, as well as GLUT2 and GLUT3 that are highly expressed in pyramidal cells located in the CA3 and dentate gyrus^5,64,65^. In addition, GLUT4 is an insulin-sensitive glucose transporter and exhibits overlapping distributions with the insulin receptor and IGF-1 receptor in the hippocampus of the rodent brain^9,66^. Here, we observed a significant reduction in expression levels of GLUTs 1, 2, 3, and especially GLUT4 in the hippocampus of diabetic rats compared to untreated diabetic rats. Among various GLUT levels evaluated, GLUT4 were significantly decreased in the brain of diabetic rats. Since the production of insulin in diabetic rats is less, the insulin-dependent GLUT4 is reduced more than other GLUTs in this condition. Among various GLUT levels evaluated, GLUT2 expression showed the lowest decrease in hyperglycemic condition, which could be due to the high sensitivity of this glucose transporter to glucose concentration and a compensatory mechanism for severe depletion of other GLUTs. These results are in line with previous reports. In this regard, Bakirtzi et al. in 2009 showed that GlUT4 in the brain is sensitive to insulin level and neuronal insulin resistance stimulates GLUT4 translocation to the plasma membrane to promote glucose uptake^67^. Sun et al. in 2018 revealed that the expression levels of GLUT3 decreased in adult hippocampal stem cells affected by STZ, which reduced their differentiation potential^68^. Moreover, Choi et al. in 2019 reported that GLUT1 expression level in the hippocampus of mice administered with STZ and fed with a high-fat diet was decreased, while they showed that hippocampal expression of GLUT2 was increased in diabetic rats^69^.

In this study, QC and QCSPIONs were able to increase the expression of GLUT2, GLUT3, and GLUT4 up to normal levels. Due to the direct effect of QC on the regeneration of pancreatic beta cells and subsequent insulin production, the expression of GLUT4 increased more than other GLUTs under QC treatment. Our results are in agreement with reports that have indicated that QC supplementation and other flavonoids can greatly regulate the expression level of GLUTs in the brain. In this regard, Eid et al. in 2015 showed that QC treatment in culturing L6 skeletal muscle cells resulted in the transfer of GLUT4 to the cell surface and increase glucose uptake by activation of AMPK^70^. Mehta et al. in 2017 showed that chronic stress decreased the expression of insulin receptor and GLUT4 in the hippocampus of mice. QC improved the disruption of the insulin signaling pathway and increased GLUT4 expression and insulin receptors of the hippocampus^71^. In the same year, Sandeep et al. showed that expression levels of GLUT1, GLUT2, GLUT3, and GLUT4 were reduced in the brain of diabetic rats. QC supplement was able to greatly increase the level of GLUTs and the main components involved in the insulin signaling pathway^72^. Besides, Eldamarawi et al. in 2020 showed that cotreatment of QC with metformin reduced hyperglycemia and insulin resistance by increasing the expression of GLUT-4 through neutralizing oxidative stress and inflammatory states in skeletal muscles and adipose tissue of diabetic rats^73^.

Moreover, the results of our study indicate that the expression of hippocampal IGF-1 in the diabetic group is significantly lower than the control group. These results are in accordance with a study by Ola et al. that reported the downregulation of IGF-1 in the brains of diabetic rats^14^. Furthermore, Zebrowska et al. reported that serum concentration of IGF-1 significantly reduced in patients with T1D^74^. A number of studies suggest that insulin has a regulatory role in the synthesis of IGF-1. Through an upregulation of hepatic growth hormone (GH) receptor expression, insulin increases the hepatic sensitivity for GH stimulation in the production of IGF-1. Insulin also increases IGF-I mRNA concentrations directly by increasing transcript stability^75,76^. Therefore, decreased expression of IGF-1 can be attributed to insulin deficiency in diabetes. IGF-1 induces growth, proliferation, differentiation, survival, and stability of neurons, as well as stimulates neuronal glucose uptake^77,78^. The IGF-1 signaling pathway is also responsible for controlling the phosphorylation of tau protein. The decrease in IGF-1 and disruption of the signaling pathway lead to tau hyperphosphorylation and neuronal death^79^. On the other hand, reducing the expression of IGF-1 under hyperglycemic conditions led to a decrease in the glucose uptake through GLUT4 in neurons by affecting on translocation of this glucose transporter^78^.

Here, IGF-1 mRNA level was increased in diabetic rats after oral administration of QC and QCSPIONs. Our results are in agreement with reports which have indicated that flavonoids can greatly regulate the expression level, synthesis, and secretion of neurotrophic factors in the brain. In this regard, Ola et al. demonstrated that treatment with morin increases the expression level of IGF-1 in the brain of diabetic rats^14^. Isik et al. also showed that level of IGF-1 increased after treatment with curcumin in diabetic rats^80^. To the best of our knowledge, there is no report evaluating QC on IGF-1 expression level.

## Conclusion

In general, our results showed that hyperglycemia can dysregulate the miR-29 family that may be the reason for the downregulation of the genes involved in glucose transport. Because in silico analysis in this study and experimental results in other studies have shown that IGF-1 and GLUTs are the direct targets of the miR-29 family, it can be concluded that miR-29 family dysregulation may be a reason for the downregulation of these genes. QC in pure and particularly conjugated with SPIONs leads to normalization level of these microRNAs and consequently the target genes. Based on the results, we propose that oral treatment of QCSPIONs can improve glucose homeostasis disorder via regulation of miR-29 family expression and increasing GLUTs and IGF-1 gene expression levels. Overall, conjugation of flavonoids such as QC with superparamagnetic nanoparticles can be a promising clinical path against CNS complications caused by diabetes in the future.

## Methods

### Animals

Male Wistar rats weighing 200–230 g (40 animals) were bought from the Royan Institute (Isfahan, Iran) and housed for 2 months at 25 °C ± 2 °C with 40–50% humidity under a 12 h light/dark cycle with free access to food and water. All maintenance, handling, experiments, and tissue collection were performed following the guidelines for the care and use of laboratory animals (USA National Institute of Health Publication No 80–23, revised 1996) and in compliance with the ARRIVE guidelines (http://www.nc3rs.org.uk/page.asp?id=1357) and were approved by the animal ethics committee of the University of Isfahan.

### Induction of diabetes and experimental design

Diabetes induction and experimental design were elaborated by our colleagues in a previous study^53^. Briefly, T1D was induced by intraperitoneal injections of 20 mg kg^−1^ STZ on 5 consecutive days. In order to confirm the incidence of T1D in the rats, blood samples were taken from the tail vein after the last injection of STZ and glucose levels measured by a glucometer. All STZ-treated rats revealed fasting glycemia over 250 mg dL^−1^ and are used in our study.

All rats were randomly divided into five groups (8 animals each):

Control group: Rats in the control group receive deionized water (DI) for 35 days.

Diabetes group: Rats in the diabetic group receive deionized water (DI) for 35 days.

QC group: Rats in the diabetic group treated with 25 mg kg-1 of free QC solution for 35 days.

SPIONs group: Rats in the diabetic group were treated with 25 mg kg-1 Fe_3_O_4_ NPs for 35 days.

QSPIONs group: Rats in the diabetic group were treated with 25 mg kg-1 QCFe_3_O_4_ NPs for 35 days.

All of the treatments were dissolved in deionized water (DI) immediately before administration and gavaged in separate groups of rats 5 days after the last injection of STZ. The dose and duration of quercetin were selected based on previous studies that revealing beneficial effects of quercetin on diabetic complication^81–84^. In order to evaluate the effect of treatments on the blood glucose levels, the blood glucose levels were measured seven times: two times before the onset of treatment, the onset of treatment, and four times after the treatment at intervals of ten days. At the end of the experiment, all animals were sacrificed by ketamine-xylazine anesthesia (100 mg/kg ketamine,10 mg/kg xylazine). The hippocampus was removed from the hemispheres and kept at − 70 °C until use.

### Synthesis of QCSPIONs

QCSPIONs were synthesized by our colleagues in a previous study. In summary, the synthesis of dextran-coated superparamagnetic iron oxide nanoparticles was performed by using the chemical co-participation (CPT) method. For this purpose, 1.135 gr of anhydrous FeCl_3_ with 0.695 gr of FeCl_2_ was dissolved in 200 mL DI water. After complete mixing, some ammonia solution was added to the mixture at 70 °C and stirred for 1 h until the pH of the solution reached nine. In the next step, 0.45 gr of dextran was dissolved in 50 mL water and then added to the above mixture. The mixture was stirred continuously for two hours at 90 °C and then by an external magnet, the resultant dextran-coated superparamagnetic iron oxide nanoparticles were collected. After washing with DI water and ethanol, the nanoparticles were dehydrated in an oven at 70 ºC overnight. To prepare QCSPIONs, QC was added to dextran-coated superparamagnetic iron oxide nanoparticles and EDC/NHS was used as a linker. Then, synthesized QCSPIONs were sequestered from suspension by a magnet. After washing with DI water, and acetone and QCSPIONs were dehydrated using a freeze drier.

### RNA extraction

To extract RNA, 50–100 mg of hippocampus tissue was used for each sample. Tissue samples were squashed in a sterile petri dish, and then total RNA (including messenger RNA and microRNA) was extracted using TRIzol (Invitrogen, Life Technologies, Grand Island, NY, USA) according to the manufacturer’s protocol. The destructed tissue in TRIzol solution was completely homogenized by repetitive pipetting using a syringe 2.5 cc with a needle 21 g. The quantity and quality of extracted RNA were assessed using a nanodrop spectrophotometer (Thermo Fisher Scientific, USA). The integrity of the extracted RNA was checked by 18 s and 28 s ribosomal bands on 1% agarose gel, stained with GelRed, without the presence of extra bands. Before the formation of cDNA, 1 μg of the extracted RNA was treated with 1 U RNase-free DNase I (Thermo Fisher Scientific Inc, USA) to remove the contamination of DNA in RNA samples. All steps of the experiment were performed under a laminar hood and by using RNase-free gloves, tubes, and strips to prevent RNA degradation.

### cDNA synthesis

500 ng treated RNA with DNase I, was used for cDNA synthesis using a cDNA synthesis kit (Takara, Japan). The reaction in a final volume of 10 μL containing 500 ng RNA, 2 μL 5 × prime script buffer, 0.5 μL RT enzyme, 0.5 μL oligo dT primer, 0.5 μL random 6mer was incubated at 37 °C for 15 min, and at 85 °C for 5 s.

Moreover, cDNA synthesis for microRNA was performed using BON-miR miRNA 1st-strand kit (Bonyakhteh, Tehran, Iran, Cat No # BON209001) through polyadenylation. Briefly, elongation of microRNAs was performed in a polyadenylation reaction, with a final volume of 20 μL at 37 °C for 30 min. Then the reaction to make cDNA was performed immediately after polyadenylation reaction and using the existing compounds in BON-miR miRNA 1st-Strand cDNA Synthesis Kit, for each sample of polyadenylated RNA.

### Real-time PCR

Gene transcripts were measured in Real-time PCR, using 2 × Master Mix Green (Ampliqon Odense, Denmark). In order to normalize levels of mRNA and microRNA expression, β-actin and Snord-47 genes were selected as reference genes respectively. As the expression level of these genes is stable relative to changes in glucose and stable in different tissues, they are good choices for analysis of mRNA and microRNA expression. Primer genes of this study were designed using the online software of OligoArchitect (www.oligoarchitect.com/LoginServlet), and then their specificity was examined on the NCBI website (www.NCBI. nlm.nih.gov/blast). The best primers were selected and purchased from Bioneer Company (City, Korea). Sequences of the primers used in this study are listed in Table 2. qPCR reaction to measure the expression level of mRNA was performed in a final volume of 10 μL, containing 5 μL SYBR Green 1 μL cDNA, 0.5 μM of 10 pM forward, 0.5 μM of 10 pM reverse primers. The reaction performed under the condition of 15 s at 95 °C as the first denaturation step, followed by 39 cycles at 95 °C for 15 s and 57 °C for 30 s, and 72 °C for 15 s.

**Table 2:** Primers for real-time PCR.

Target bp^a^	Forward primer 5′ → 3'	Reverse primer 5′ → 3'	Amplicon bp^a^
β-actin	CTCTATGCCAACACAGTG	AGGAGGAGCAATGATCTT	123
GLUT1	TATCAGCCACTCTCCTAT	GCCATTGTTCAGTATTCG	158
GLUT2	CATTTCAGTCCTTTGTATTGT	GATTGGTCCAGTTTATTAGATT	126
GLUT3	TTATTGTGGCTGAACTCT	AAGGTGAAGATTAGGAAGAA	181
GLUT4	TCATCTTCACCTTCCTAA	CCTCAGTCATTCTCATCT	151
IGF-1	GGTTGATGAATGGTTCCTTA	GCCTAGACGAGATTATTAGATT	81

Forward and reverse primers for microRNA were designed and synthesized by Bonyackteh Company (Bonyakhteh, Tehran, Iran). qPCR reaction was carried out in a final volume of 13 μL, containing 1 μL cDNA, 0.5 μL miRNA specific forward primer, 0.5 μL universal reverse primer, 6.5 μL qPCR master mix. After the initial denaturation step at 95 °C for 2 min, the reaction was performed as 40 cycles at 95 °C for 5 s and 60 °C for 30 s. The development of PCR steps was specified by measuring the intensity of light emitted from SYBER Green at the end of the elongation phase. At the end of the reaction, the specificity of products for PCR reaction was confirmed by analyzing the melting curves. The concentration of primers, cDNAs, the efficiency of primers, and conditions of PCR was optimized. Gene expression was calculated using the 2^−ΔΔCT^ method.

### Statistical analyses

The results were reported as mean ± SEM and analyzed by using the GraphPad Prism software package (GraphPad Software Inc., San Diego, CA, USA). Groups were compared by one-way ANOVA followed by Tukey’s multiple comparison tests or Student’s t-test. Value of p < 0.05 was considered to be statistically significant.
